# Global Context-Aware-Based Deformable Residual Network Module for Precise Pest Recognition and Detection

**DOI:** 10.3389/fpls.2022.895944

**Published:** 2022-06-02

**Authors:** Lin Jiao, Gaoqiang Li, Peng Chen, Rujing Wang, Jianming Du, Haiyun Liu, Shifeng Dong

**Affiliations:** ^1^National Engineering Research Center for Agro-Ecological Big Data Analysis and Application, Information Materials and Intelligent Sensing Laboratory of Anhui Province, School of Internet, Anhui University, Hefei, China; ^2^Institute of Intelligent Machines, Hefei Institutes of Physical Science, Chinese Academy of Sciences, Hefei, China; ^3^Institutes of Physical Science and Information Technology, Anhui University, Hefei, China; ^4^Science Island Branch, University of Science and Technology of China, Hefei, China

**Keywords:** deep learning, convolutional neural network, deformable residual network, agricultural pest, target detection

## Abstract

An accurate and robust pest detection and recognition scheme is an important step to enable the high quality and yield of agricultural products according to integrated pest management (IPM). Due to pose-variant, serious overlap, dense distribution, and interclass similarity of agricultural pests, the precise detection of multi-classes pest faces great challenges. In this study, an end-to-end pest detection algorithm has been proposed on the basis of deep convolutional neural networks. The detection method adopts a deformable residual network to extract pest features and a global context-aware module for obtaining region-of-interests of agricultural pests. The detection results of the proposed method are compared with the detection results of other state-of-the-art methods, for example, RetinaNet, YOLO, SSD, FPN, and Cascade RCNN modules. The experimental results show that our method can achieve an average accuracy of 77.8% on 21 categories of agricultural pests. The proposed detection algorithm can achieve 20.9 frames per second, which can satisfy real-time pest detection.

## Introduction

Automatic insect recognition has attracted more and more attention in the field of agricultural engineering. Conventional pest management in farmland has relied mainly on periodic spraying plans based on schedules. With the increasing attention to environmental impact and pest control cost, integrated pest management (IPM) (Bernardo, 1993) has become one of the most effective and accurate pest management methods. It abandons the conventional spraying procedure and depends more on the actual existence or possibility of field insects. The use of insect attractants and traps is commonly adopted to monitor agricultural pest in the farmland. Growers and IPM consultants regularly monitor the pest situation of farmland by manually counting harmful insects on traps, and control agricultural pests according to specific insect distribution. However, it is time-consuming and inefficient. Therefore, automatic identification and counting of pests is the important step of IPM, which makes a major contribution for producers with large farmland.

As described in the study by Guo et al. (2021), the process of frequently used automatic recognition and counting methods can be described as follows: collecting insect pest images using trapping devices followed by automated counting *via* computer vision-based detection methods. Thus, the precise pest detection will be decided by computer vision-based detection algorithms. Wen and Guyer (2012) developed image-based orchard insect identification and classification methods by using the local features model, global features model, and the combination model, respectively. The method is more robust and can work on field insect images considering the messy background, missing insect features, and varied insect size and pose. Because each target of the sample case has different colors and distinctive body shapes, Hassan et al. (2014) proposed an automatic insect identification framework that can identify grasshoppers and butterflies by manipulating insects’ color and their shape feature. Yalcin (2015) used multiple feature descriptors, i.e., Hu moment, elliptic Fourier descriptors, radial distance function, and local binary patterns, to identify and classify the insect images under complex background and illumination conditions. We know that the insect pest recognition accuracy of traditional approaches heavily depends on the hand-designed features by various algorithms. However, precise and proper features need to be carefully designed and selected for high accuracy, leading to expensive works and expert knowledge. It will be even worse when the background is complex.

Convolutional neural networks (CNNs) are effective in the fields of image recognition and classification due to the powerful ability of feature extraction. The framework of region-based CNN was developed to improve the detection accuracy (Girshick et al., 2014). CNN modules were used to automatically extract the feature representations from images, ignoring hand-crafted features. Two-stage object detection methods are the mainstream detection framework (Lin et al., 2017a; Ren et al., 2017; Cai and Vasconcelos, 2018). Specifically, the region proposal generation algorithms, such as Selective Search (Uijlings et al., 2013), EdgeBox (Zitnick and Dollár, 2014), and RPN (Ren et al., 2017), AF-RPN (Jiao et al., 2020), are applied to generate a set of region candidates (region of interests, ROIs) in the first stage, and then, these region proposals are used for obtaining multi-class labels and refining the bounding boxes using the R-CNN network. CNN-based object detection algorithms have been applied to pest detection in precision agriculture. Gomez Selvaraj et al. (2019) use Faster R-CNN detector with ResNet50, InceptionV2, and single-shot detector (SSD) with MobileNetV1 to detect banana disease and pest, and detection results show that deep CNN is a robust and easily deployable strategy for banana pest recognition. He et al. (2020) used a two-stage detection framework, Faster RCNN, to detect brown rice plant hopper, and compared it with a one-stage detection method, YOLO V3 (Redmon and Farhadi, 2018). Experimental results demonstrate that the performance of the two-stage detection algorithm significantly outperforms the one-stage detector. Wang et al. (2021) proposed a sampling-balanced region proposal network (S-RPN) and attention-based deep residual network for detecting multi-classes pests with a small size, achieving good performance compared with other state-of-the-art detectors. Jiao et al. (2020) developed a two-stage end-to-end agricultural detection method named AF-RCNN to recognize and localize multi-classes pest targets, achieving 56.4% mAP and 85.1 mRecall on a 24-types pest dataset. However, there are pose-variant, serious overlap, dense distribution, and interclass similar pests in our experimental dataset, leading to poor performance of pest feature extraction. Thus, the accurate and robust pest detection system still faces great challenges.

The hypothesis of this study is that the features of agricultural pests can be obtained by machine learning through images analysis, while they traditionally need professional knowledge of the expert. However, deep learning-based pest detection methods still face some challenges according to the aboded description. For example, there are pose-variant, serious overlap, dense distribution, and interclass similar pests in our experimental dataset, leading to poor performance of pest feature extraction. Thus, the accurate and robust pest detection system still faces great challenges. It is necessary to propose a new method to address the precise recognition of pest with pose-variant, serious overlap, dense distribution, and interclass similar pests. A deep CNN is applied to automatically extract rich feature information from pest images with multi-pose, high similarity, and high overlap. A feature extractor module is used to enhance the features of region-of-interest of pest by merging the global information of pest image. The objectives of this work are to (1) develop a deformable residual block (DRB) network to extract detailed feature information of multi-class pest with pose-variant, serious overlap, dense distribution, and interclass similar pests; (2) propose a global context-aware module to get high-quality feature of region-of-interests of pests; and (3) introduce an end-to-end two-stage pest detection algorithm to accomplish the identification and detection of 21-types of agricultural pest.

## Materials and Methods

In this part, the whole framework of our agricultural pest detection network is first demonstrated. Second, the materials used in this study are presented. Third, the proposed DRB network (DRB-Net) is described in detail. Finally, the region proposal generation algorithm and the global context-aware feature extraction module are introduced, respectively.

### Agricultural Pest Detection Framework

**FIGURE 1:**
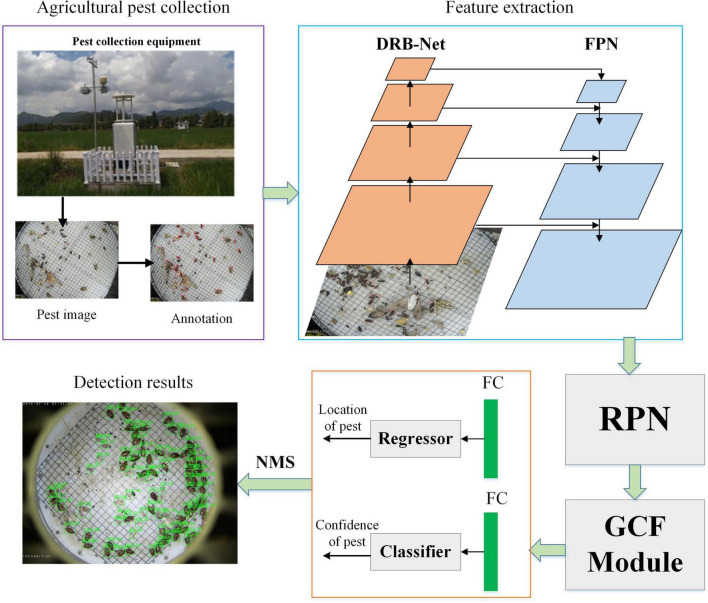
Whole framework of agricultural pest detection. FC represents the fully connected layer.

In this part, the overview of the whole detection framework is shown in Figure 1. A pest collection equipment is used to obtain a large number of pest images and then these pest images are labeled by professional experts. Pest images are input into DRB-Net for extracting deformable feature information, and feature pyramid network (FPN) is applied to extract multi-scale fusion pest features. These extracted features are input to region proposal network (RPN) to generate a set of pest proposals, and then a global context-aware feature (GCF) extractor is developed to produce region-of-interest (RoI) with global context information. Following R-CNN (Girshick et al., 2014), two-stage CNNs are used for specific-class classification and localization of each RoI *via* an end-to-end way. Finally, the NMS (Non-Maximum Suppression) algorithm (Rosenfeld and Thurston, 1971) is adopted to filter redundant bounding boxes, and obtain pest detection results.

### Materials

In this study, the experimental images are collected by an automatic device that uses a multispectral light trap for attracting crop pests. HD camera above the tray of this device is set to take images, which were saved in a JPG format with 2,592×1,944 pixels. In this work, the width and height of the pest images are resized to 800×600 for high efficiency. The dataset contains 24,412 images and 21 types of pests. Table 1 shows details of our collected agricultural pest dataset, including the scientific names, the pest images, the number of pest instances and pest images, and the average relative scale of each pest instance. In order to train and evaluate the performance of the CNN-based objector, all pest images are randomly split into train set (15,378 images), validation set (6,592 images), and test set (2,442 images), respectively.

**TABLE 1:** Details of 21 types of agricultural pest, including the pest images, number of pest instances of each category, number of pest images of each category, and the average relative scale of each pest instance.

Classes	Image	Number of instances	Number of pest images	Average relative scale (%)
*Cnaphalocrocis medinalis* (CM)	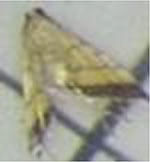	1,224	932	0.1214
*Chilo suppressalis* (CS)	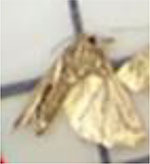	1,285	454	0.1793
*Mythimna separate* (MS)	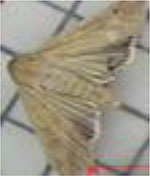	8,374	3,637	0.3978
*Helicoverpa armigera* (HA)	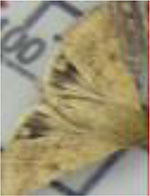	26,588	8,740	0.2814
*Pyrausta nubilalis* (PN)	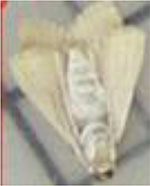	15,739	5,294	0.2267
*Athetis lepigone* (AL)	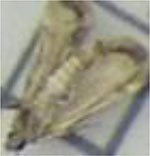	28,932	7,200	0.1298
*Spodoptera litura* (SL)	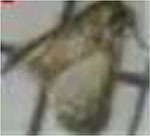	1,896	1,543	0.4572
*Spodoptera exigua* (SE)	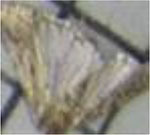	7,116	3,527	0.1377
*Sesamia inferen* (SI)	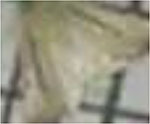	1,768	1,335	0.2776
*Agrotis ypsilon* (AY)	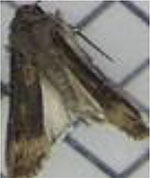	3,890	2,314	0.5703
*Mamestra brassicae* Linnaeus (MbL)	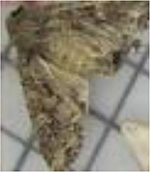	2,170	1,632	0.4259
*Scotogramma trifolii* Rottemberg (StR)	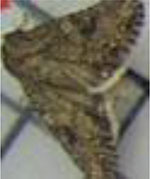	4,393	3,051	0.2816
*Agrotis segetum* (AS)	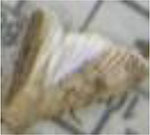	1,615	1,330	0.4024
*Agrotis tokionis* Butle (AtB)	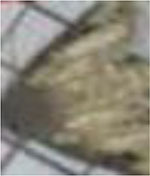	465	351	0.6375
*Holotrichia oblita* Faldermann (HoF)	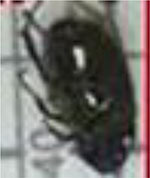	82	70	0.3348
*Holotrichia parallela* (HP)	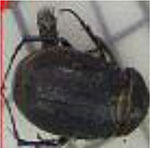	11,325	3,002	0.2518
*Anomala corpulenta* (AC)	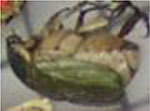	52,134	5,083	0.2466
*Gryllotalpa orientalis* Burmeister (GoB)	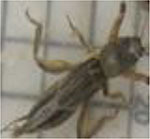	6,480	3,589	0.9530
*Pleonomus canaliculatus* (PC)	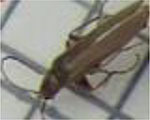	157	109	0.3281
*Agriotes subrittatus* Motschulsky (AsM)	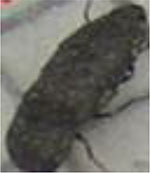	6,161	1,729	0.1129
*Melanotus caudex* Lewis (McL)	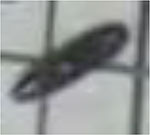	677	224	0.1584

**FIGURE 2:**
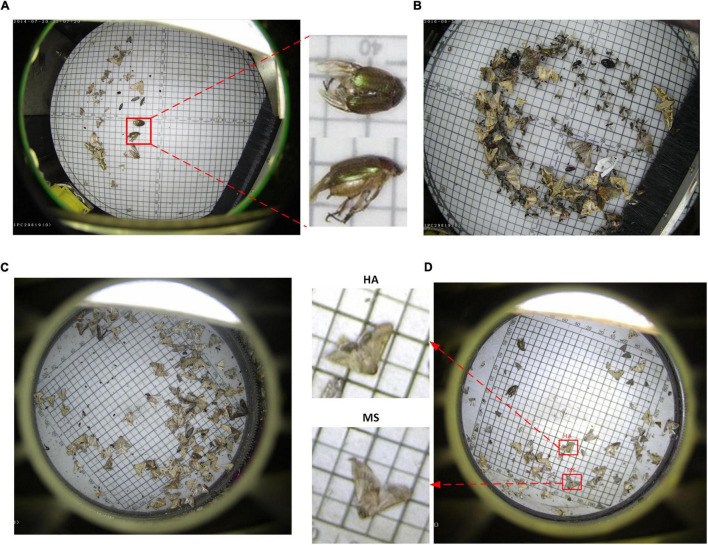
Some examples of agricultural pest images. **(A)** Different shapes of the same class of pests. **(B)** Serious overlap. **(C)** Dense distribution. **(D)** High similarity between the classes “HA” and “MS.”

To recognize the object of an image using deep CNN, the class and localization of each pest instance needs to be labeled. In this study, these pest instances are hand-annotated by several pest experts using LabelImg software, which is provided by the Computer Science and Artificial Intelligence Laboratory at MIT. Generally, rectangular bounding boxes are used to annotate the location of a pest instance, which can be represented as (*x*_1_,*y*_1_,*x*_2_,*y*_2_), here (*x*_1_,*y*_1_) is the coordinate of top-left and (*x*_2_,*y*_2_) is the coordinate of bottom-right. Figure 2 shows some examples of agricultural pest images. Pose variations of the same types of pest will decrease the precise recognition, as presented in Figure 2A. Besides, the distribution of pest targets is seriously dense and worse is that the pest targets are overlapped, as shown in Figures 2B,C, respectively. The appearance of two different categories of pest has a high similarity, for example, the class “HA” and “MS,” as shown in Figure 2D.

### Deformable Residual Block Network

As we know, a deep residual network is a common backbone for extracting features. For ResNet50 (He et al., 2016), it contains 16 residual blocks with 50 convolutional layers. The output feature map of each residual block in ResNet50 network has different resolutions. The details of the ResNet50 are reported in Table 2. For the same class pest instances with different poses and shapes, the common backbone cannot effectively extract the feature information of pest, leading to poor recognition of pest with different shapes and poses.

**TABLE 2:** Description of standard ResNet50.

Layer name	Setting of convolutional layers
Conv1	7×7, 64, stride 2
	3×3 max pool, stride 2
Conv2_x (block 1)	$\left[\begin{array}{c} 1\times 1,\;64\\ 3\times 3,\;64\\ 1\times 1,\text{ 256} \end{array}\right]\times 3$
Conv3_x (block 2)	$\left[\begin{array}{c} 1\times 1,\;128\\ 3\times 3,\;128\\ 1\times 1,\text{ 512} \end{array}\right]\times 4$
Conv4_x (block 3)	$\left[\begin{array}{c} 1\times 1,\;256\\ 3\times 3,\;256\\ 1\times 1,\;1,\text{ 024} \end{array}\right]\times 6$
Conv5_x (block 4)	$\left[\begin{array}{c} 1\times 1,\;512\\ 3\times 3,\;512\\ 1\times 1,\;2,\text{ 048} \end{array}\right]\times 3$
	Average pooling, 7×7, stride 1

**FIGURE 3:**
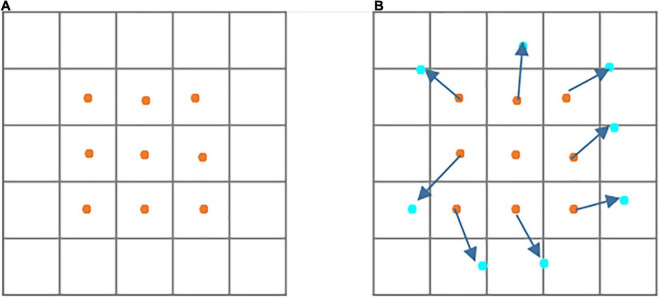
Illustration of sampling location of traditional and deformable convolutions. **(A)** Regular sampling of traditional convolution. **(B)** Irregular sampling (indicated in deep blue arrows) of deformable convolution.

Inspired by previous work (Dai et al., 2017), it is known that deformable convolution can enhance the capability of CNNs of modeling geometric transformation of objects. The difference between traditional convolution and deformable convolution can be shown in Figure 3. It shows that the sampling locations of deformable convolution are irregular compared with the regular sampling of traditional convolution.

Additionally, from the aspect of mathematical description, the standard convolution can be defined as following:


(1) $$\text{y}\,\left(p_{0}\right)=\sum_{p_{n}\in R}w\,\left(p_{n}\right).x\,\left(p_{0}+p_{n}\right)$$

where y(*p*_0_) denotes the output feature map for each location *p_0*; *ℛ* represents the sampling space in the input feature map *x*; *w* is the learnable weight; *p_n* enumerates the location of sampling space *R*.

However, in deformable convolution, the sampling space is enlarged by adding the offsets, which can be defined by Equation (2):


(2) $$\text{y}\,\left(p_{0}\right)=\sum_{p_{n}\in R}w\,\left(p_{n}\right).x\,\left(p_{0}+p_{n}+△\,p_{n}\right)$$

where △*p*_*n*_ denotes the offset, which can be obtained by network learning. However, △*p*_*n*_ is typically fractional. The bilinear interpolation operation is used for obtaining the final offsets.

**FIGURE 4:**
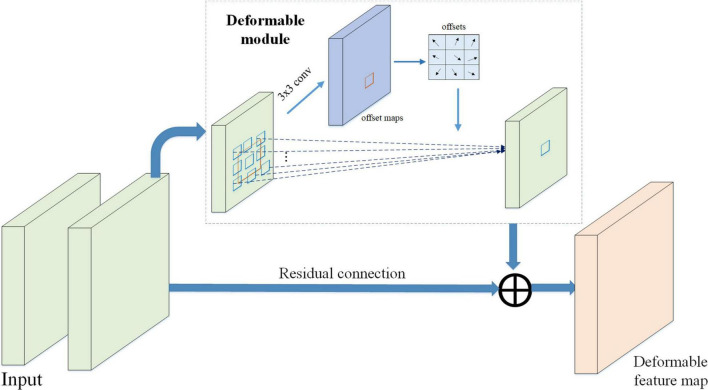
Architecture of the deformable residual block.

Therefore, to detect pose-invariant and shape-invariant pest instances, a deformable convolution module has been embedded into the deep residual network, which can extract multi-scale and deformable pest features. The architecture of DRB is presented in Figure 4. The deformable module is designed for extracting shape information of pest. Finally, the DRB is introduced into the residual blocks of ResNet50 backbone, achieving the effective extraction of deep deformable pest feature information.

As we know that low-level features usually have large spatial size and more-grained detail information, while high-level features tend to contain more semantic knowledge. Generally, low-level features are beneficial for the detection of small objects. To identify pest with different sizes, a multi-scale feature extraction network, i.e., FPN (Lin et al., 2017a) is adopted to fuse pest feature information from low-level and high-level feature maps.

### Generation of Pest Region Proposal

**FIGURE 5:**
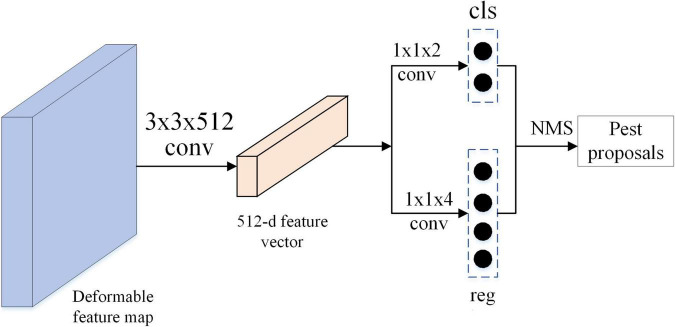
Network structure of pest region proposal generation module.

In Faster RCNN (Ren et al., 2017), Ren et al. (2017) proposed the RPN to generate a set of region proposals. This region proposal is the region that contains the object instance. As shown in Figure 5, RPN model consists of two fully connected layers: classification layer and regression layer. The former outputs 2*k*-dimension vector encoding the classification confidence (objects or not objects), and the latter outputs 4*k*-dimension vector encoding the coordinates of bounding box. In this study, *k* denotes the number of anchor boxes in RPN. The parameter *k* is set to 1, leading to fewer parameters of RPN and improving the efficiency without decreasing the quality of pest region proposals. The stochastic gradient descent (SGD) (LeCun et al., 1989) method was used for end-to-end training, which allowed the convolutional layers to be shared between the RPN and the Fast R-CNN components. The feature maps from deformable FPN are propagated forward to pest proposal generation network, and then a set of pest proposals with classification scores and coordinates of bounding boxes is received as output.

However, these pest proposals may be reductant and of low quality. Generally, the NMS algorithm is adopted to decrease the overlapped bounding box candidates and improve the quality. Given a series of proposals with classification scores in an image, the IoU ratios between the bounding box with the highest score and its neighboring bounding boxes are calculated. The scores of neighboring bounding boxes will be suppressed when their IoU ratios are lower than the preset values. The process of NMS can be described mathematically as Equations (3 and 4):


(3) $$s_{i}=\left\{ \begin{array}{cc} s_{i} & I\,o\,U\,\left(B,b_{i}\right)<t\\ 0 & I\,o\,U\,\left(B,b_{i}\right)\geq t \end{array}\right.$$

(4) $$I\,o\,U\,\left(B,b_{i}\right)=\frac{a\,r\,e\,a\,\left(B\,⋂b_{i}\right)}{a\,r\,e\,a\,\left(B\,⋃b_{i}\right)}$$

where B is the bounding boxes with the highest score, b_i_ represent the *i*-th neighboring boxes of B with confidence score *S_i*. *t* is the threshold value of IoU ratio, which is set to 0.7; *area*(B∩b_i_) denotes the intersection of boxes with the highest scores and their neighboring boxes, and *area*(B∩b_i_) is their union.

The low-quality bounding box candidates can be removed using the NMS algorithm. Notably, a different number of region proposals are used during training and testing. In our study, 1,000 proposals are selected according to their scores for network training and testing. Besides, the effect of different numbers of proposals is explored in the section of experiments.

### Global-Context Feature Module

**FIGURE 6:**
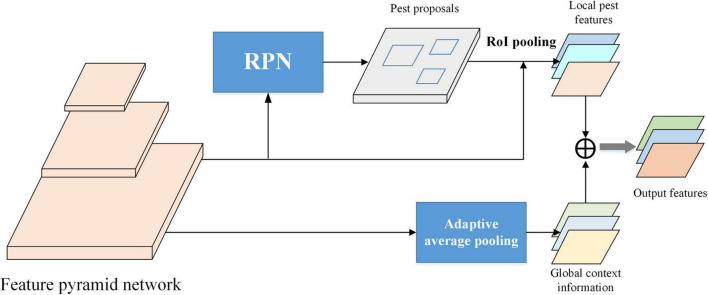
Description of global context-aware feature module.

For the challenging scenarios in agricultural pest detection, such as cluttered background, foreground disturbance, simple integration of high-level, and low-level features may fail to detect the pest targets due to lacking the global context. A global context-aware feature module is designed in this work to extract rich information of agricultural pest, as shown in Figure 6. Given the full-image convolutional feature map in the FPN, the feature maps are pooled by global pooling, which can be implemented by an adaptive average pooling using the entire image’s bounding box as the RoI. The pooled features are input into the post-RoI layer to get a global context pest feature. And the global feature is concatenated with the local RoI feature developed by RoI pooling. Therefore, additional global context information is accessible for each pest proposal, improving the recognition and localization of pest under complex scenes.

### Unified Pest Detection Network

To detect the multi-categories pest, the RPN (Ren et al., 2017) and Fast R-CNN (Girshick, 2015) module are combined into a single network *via* an end-to-end way, as shown in Figure 1. These two networks can be separately trained. However, the separate training will lead to different convolutional layers. Therefore, according to the training procedure in Ren et al. (2017), joint training between RPN and R-CNN was performed, which allows for shared convolutional layers. In each SGD iteration, the forward pass generates pest proposals, which are then fed into the Fast R-CNN detector for training. The backward propagation happens as usual, and for the sharing convolutional layer, the backward propagated signals come from the combination of RPN losses and Fast R-CNN losses. Additionally, another advantage of the end-to-end training method is that it can reduce the training time compared with the separate training model.

### Evaluation Metrics

To verify the performance of our proposed agricultural pest detection method, the metrics of average precision (AP) and recall are adopted. A true positive (TP) is when the network correctly identifies the pest target. A predicted box is viewed as false positive (FP) when the model falsely identifies a pest target, for example, calling something an “*Agrotis ypsilon*” that is not an “*Agrotis ypsilon*.” The precision (P) and recall (R) are defined as follows:


(5) $$p\,r\,e\,c\,i\,s\,i\,o\,n=\frac{\#\,T\,P}{\#\,T\,P+\#\,F\,P}$$

(6) $$r\,e\,c\,a\,l\,l=\frac{\#\,T\,P}{G\,T}$$

In which #*TP*, #*FP* present the number of TP and FP, respectively. Ground Truth (GT) denotes the total number of ground truth boxes.

The average precision (AP) can be calculated based on the shape of the precision/recall curve.


(7) $$A\,P=\int_{0}^{1}P\,dR$$

The mean AP (mAP) averaged over all object classes is employed as the final measure to compare performance over all object classes, and it is defined as follows:


(8) $$m\,A\,P=\frac{1}{C}\,\sum_{j=1}^{C}A\,P_{j}$$

where *C* is the number of classes, which is 21 in this study.

Additionally, the AP0.75 denotes the AP at IoU 0.75, which is applied to evaluate the detection accuracy of pest detection. The strict metrics, for example, mean AP and Average recall (AR) across IoU thresholds from 0.5 to 0.95 with an interval of 0.05, are used to further verify the performance of the proposed method. ARs, Arm, and ARl is the average recall of small, medium, and large pest target, respectively. In this study, the small, medium, and large pest target can be defined in Table 3.

**TABLE 3:** Definition of the small, medium, and large pests.

	Min rectangle area (pixel)	Max rectangle area (pixel)
Small pest	0 × 0	32 × 32
Medium pest	32 × 32	96 ×96
Large pest	96 ×96	∞×∞

## Experimental Results and Analysis

### Experimental Details

The proposed method and other state-of-the-art models are trained using the back-prorogation algorithm and SGD method, with momentum 0.9 and initialize learning rate to 0.0025 that will be dropped by 10 at the 8-th and 11-th epoch followed by Ren et al. (2017). The batch size is set to 4 during training. The proposed detection module is trained *via* an end-to-end way. These experiments are performed on a dell T3630 computer workstation with NVIDIA TITANX, 24G graphics card, and Intel core i9-9900K. Deep CNN was built based on Pytorch framework under Ubuntu18.02 operating system.

### Comparison Results of Each Category of Agricultural Pest

Table 4 reports the detection results. It presents the AP of 21 pest classes performed by our method and other state-of-the-art models. Table 4 suggests that that our method can achieve more precise recognition accuracy on all the categories. It is obvious that the proposed method significantly outperforms one-stage detectors, for example, 6.0% improvements for SSD (Liu et al., 2016), 10.0% improvements for YOLO (Redmon and Farhadi, 2018), and 13.1% improvements for RetinaNet (Lin et al., 2017b), and 7.8% inprovements for YOLOF (Chen et al., 2021). Additionally, the detection accuracy of our method is also higher than the multi-stage methods [e.g., FPN (Lin et al., 2017a) and Cascade RCNN (Cai and Vasconcelos, 2018)]. Specifically, it improves 5.3 points and 5.5 points compared with FPN and Faster RCNN, respectively.

**TABLE 4:** Detection results (AP) compared with other methods on pest dataset (unit: %).

	Method						
Class	SSD	YOLOv3	RetinaNet	FPN	YOLOF	Cascade RCNN	Our method
CM	68.7	64.7	68.2	70.0	63.9	69.6	**78.1**
CS	69.7	73.6	73.1	74.7	71.6	76.5	**80.0**
MS	79.7	77.3	75.3	82.3	79.6	82.0	**85.4**
HA	91.1	87.1	88.1	90.5	88.8	90.3	**91.6**
PN	79.6	77.0	76.7	82.0	79.9	82.7	**85.4**
AL	72.0	69.3	62.8	74.7	72.7	73.8	**78.9**
SL	81.4	73.8	78.3	83.2	81.8	84.2	**85.9**
SE	53.1	47.1	48.1	57.2	53.8	56.2	**64.9**
SI	77.1	73.0	79.1	82.6	76.9	81.5	**85.2**
AY	89.2	84.5	83.7	89.2	86.8	89.2	**91.4**
MbL	66.9	54.3	57.6	67.8	64.5	69.7	**77.0**
StR	58.2	55.6	52.5	61.4	55.8	59.9	**68.8**
AS	63.8	53.8	42.5	60.9	46.1	58.8	**68.2**
AtB	60.0	48.0	44.7	53.1	60.3	53.8	**64.0**
HoF	3.0	0.0	7.3	4.2	0.0	0.0	**16.8**
HP	93.0	89.4	87.8	90.8	88.8	90.8	**92.1**
AC	95.8	89.1	89.3	90.7	88.4	90.7	**91.6**
GoB	97.3	97.5	98.2	97.5	98.4	97.6	**97.6**
PC	54.2	44.1	43.4	53.1	42.0	52.7	**56.7**
AsM	79.0	81.6	75.2	81.9	74.9	82.0	**86.5**
McL	74.7	83.2	27.6	74.0	73.3	76.8	**87.5**
Average	71.8	67.8	64.7	72.5	70.0	72.3	**77.8**

*The detection results of our method are shown in bold.*

However, Table 4 also shows that the detection accuracy of the pest “HoF” is only 16.3%, which largely falls behind other categories of pests with adequate samples. This is because the number of samples of the pest “HoF” is only 70, leading to insufficient learning during network training. Therefore, the number of pest samples will significantly affect the detection results.

Table 4 summarizes that the “HoF” seems to be difficult to recognize on all detection models, while all the models could classify the “HA” pest. The proposed method can achieve 16.8% AP, obviously outperforming other methods. Especially, for the YOLO and Cascade RCNN detectors, the detection accuracy is 0.0%, which does not recognize this class of pests. The improvement of our method contributes to the introduction of the deformable residual network and global feature extractor, which can extract rich global pest features in deformed pest images.

### Compared Results Evaluated by Strict Metrics

The stricter standards (e.g., AP0.5:0.9, AP0.75, and AR) are applied to evaluate the detection results. The AR is used to evaluate the localization accuracy of pest targets, and ARs, ARm, and ARl are the AR of small, medium, and large-scale pest, respectively. Table 5 shows the compared detection results among SSD (Liu et al., 2016), RetinaNet (Lin et al., 2017b), YOLO (Redmon and Farhadi, 2018), Cascade RCNN (Cai and Vasconcelos, 2018), FPN (Lin et al., 2017a), YOLOF(Chen et al., 2021), and the proposed method. It is observed from Table 5 that AP@IoU [0.5:0.95] and AP@IoU = 0.75 of our method can achieve 49.6 and 58.8%, respectively, outperforming other state-of-the-art detectors. This demonstrates that our method can not only improve the accuracy of classification but also localization.

**TABLE 5:** Compared results evaluated by strict evaluation criteria.

Method	SSD	RetinaNet	YOLOv3	Cascade RCNN	YOLOF	FPN	Proposed method
AP0.5:0.9	44.2	41.2	39.6	46.4	42.1	45.9	49.6
AP0.75	51.4	48.4	42.3	54.9	47.3	53.7	58.8
AR	61.3	61.5	51.3	58.0	58.3	59.3	62.0
ARs	47.7	51.6	40.2	43.5	48.1	45.3	51.1
ARm	64.0	65.6	53.9	60.1	61.2	63.0	61.9
ARl	45.0	45.0	50.0	30.0	35.0	35.0	50.0

### Ablation Experiments

The proposed pest detection method has contributed two elements, including global-context feature (GCF) module and deformable residual block network (DRB-Net). To analyze the contribution of each component, the ablation experiments are shown in Table 6. In this study, the baseline is Faster R-CNN with FPN. We first add the GCF module to the baseline, as shown in the second row of Table 6. The DRB-Net leads to a gain of 2.5% AP. This is because of the addition of global context information, which is instrumental in the recognition of crop pest. The third row of Table 6 demonstrates that the DRB-Net can effectively boost the performance from 75.0 to 76.6%. The improvements may be result from the extraction of agricultural pest with various scales and poses. Finally, we analyze the influence of multi-scale training. From the fourth row of Table 6, we can observe that multi-scale training can improve the accuracy of pest detection. This is because the multi-scale training enhances the diversity of training samples.

**TABLE 6:** Ablation study on the major components.

GCF module	DRB-Net	Multi-scale training	mAP (%)
			72.5
✓			75.0
✓	✓		76.6
✓	✓	✓	77.8

### Detection Efficiency

Aside from detection accuracy, the detection speed also needs to be considered. Table 7 reports the results of the detection speed of the proposed method and other excellent detection models. The proposed model can run at a speed of 20.9 FPS, which outperforms Cascade RCNN (Cai and Vasconcelos, 2018). However, it underperforms other detection models, such as SSD (Liu et al., 2016), RetinaNet (Lin et al., 2017b), and YOLOv3 (Redmon and Farhadi, 2018). This is because the proposed pest detection network is a two-stage framework that uses RPN for generating pest proposals, leading to consumption of time. But one-stage detection models are proposal-free, directly regressing the bounding box of pest and classifying, resulting in higher efficiency. In summary, the precision of our method is higher than other methods, and the detection speed could satisfy the requirement of real-time detection; therefore, our method balances the pest detection efficiency and accuracy.

**TABLE 7:** Detection efficiency of agricultural pest using our method and other state-of-the-art models.

Method	Efficiency (FPS)	Accuracy
SSD	41.1	71.8
RetinaNet	21.4	64.7
YOLOv3	54.7	67.8
YOLOF	35.7	70.0
Cascade RCNN	17.2	72.3
FPN	22.0	72.5
Proposed method	20.9	77.8

### Analysis Experiments of Pest Proposals

As we know that the quality of pest proposals will decide the final detection accuracy of agricultural pest, Table 8 lists the recall of different numbers of pest proposals produced by RPN without and with DRB-Net. It shows that the quality is higher when using DRB-Net. For example, when using 50 proposals, the RPN with DRB-Net can achieve 89.0% recall, which obtains 1.4% improvements compared with RPN without DRB-Net. Thus, the introduction of DRB-Net contributes to the improvement of agricultural pest detection.

**TABLE 8:** Recalls of different number of pest region proposals generated by RPN with DRB-Net and without DRB-Net.

Number of proposals	10	50	100	1,000
With DRB-Net	55.1	89.0	95.2	95.2
Without DRB-Net	54.4	87.6	93.8	93.8

From the view of localization of pest, Table 9 shows the recalls of pest proposal produced from RPN with and without DRB-Net under different IoU thresholds while using 100 proposals. It demonstrates that the performance of RPN with DRB-Net outperforms that without using DRB-Net. With the increase of IoU, the recalls of pest proposals will gradually decrease; however, the recall of RPN with DRB-Net can achieve 13.3, obtaining 4.9% improvements than without DRB-Net. This phenomenon suggests that the DRB-Net is the main factor to promote the quality of pest proposals.

**TABLE 9:** Recalls of pest proposals generated from RPN without DRB-Net and with under different IoU thresholds.

IoU thresholds	0.5	0.6	0.7	0.8	0.9
Without DRB-Net	93.8	92.5	85.5	58.7	8.4
With DRB-Net	95.2	94.1	87.7	61.7	13.3

### Visualization of Agricultural Pest Detection Results

**FIGURE 7:**
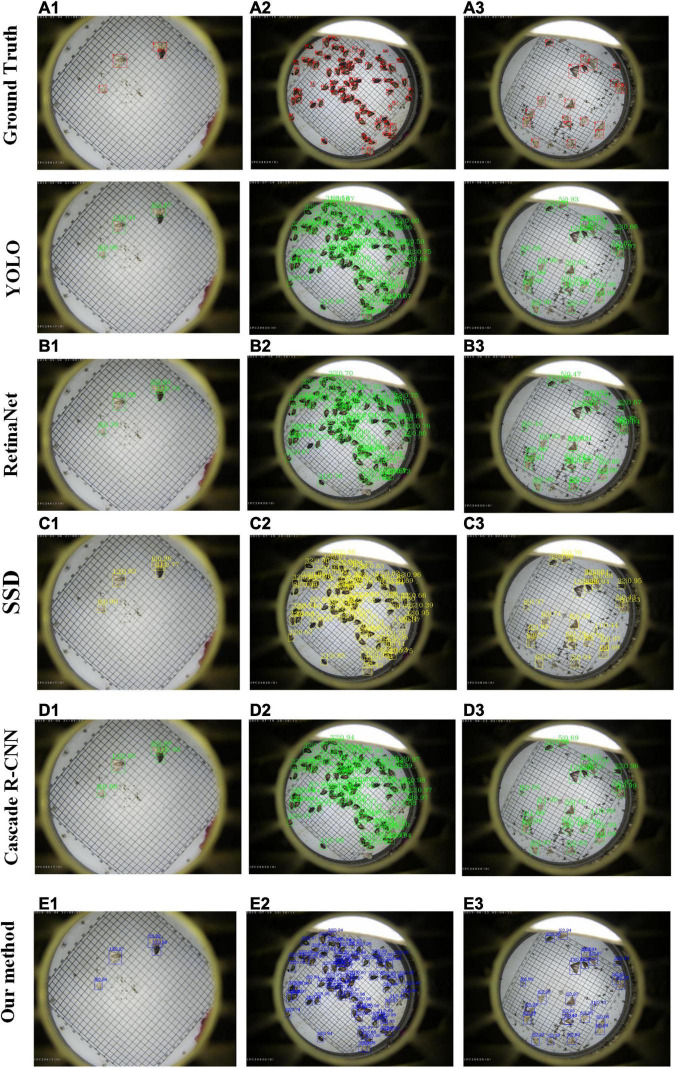
Selected examples of agricultural pest detection results by using YOLO, RetinaNet, SSD, Cascade R-CNN, and our method.

For visualization purpose, several examples of pest detection results are given in Figure 7. The row from the top to the bottom is expressed as the result of Ground truth, YOLO, RetinaNet, SSD, Cascade R-CNN, and our method. The detection results are marked by boxes with different colors. The proposed method could obtain good performance on the pest targets with sparse and dense distribution. For example, the class “HP” is undetected by using YOLO version 3 algorithm, as shown in Figure 7 (a1), while the recognition accuracy can achieve 99.0% for the proposed method, as shown in Figure 7 (d1). Additionally, for pest targets with dense distribution, our method has a higher precision of classification than other methods.

## Conclusion

As we know, insect pests are one of the main factors affecting agricultural product yield. Precise recognition and localization of insect pests benefit to timely preventive measures to decrease economic losses. However, recent pest detection methods cannot effectively recognize and localize the pest targets. In this study, a deformable residual network is developed to extract deformable feature information of crop pest. Furthermore, a global context-aware extractor is designed to obtain global features of pest images, which are combined with local features, contributing to the improvement of the detection of pest targets. Quantitative experiments were conducted on the constructed large-scale multi-class pest dataset to evaluate the performance of the proposed method, demonstrating that the proposed method outperforms other state-of-the-art detectors in the view of pest localization and classification.

## Data Availability Statement

The original contributions presented in this study are included in the article/supplementary material, further inquiries can be directed to the corresponding author.

## Author Contributions

LJ: conceptualization, methodology, software, investigation, and writing draft. GL: validation, formal analysis, visualization, and software. PC: validation and revised the manuscript. JD, RW, HL, and SD: writing and revising. All authors contributed to the article and approved the submitted version.

## Conflict of Interest

The authors declare that the research was conducted in the absence of any commercial or financial relationships that could be construed as a potential conflict of interest.

## Publisher’s Note

All claims expressed in this article are solely those of the authors and do not necessarily represent those of their affiliated organizations, or those of the publisher, the editors and the reviewers. Any product that may be evaluated in this article, or claim that may be made by its manufacturer, is not guaranteed or endorsed by the publisher.
